# Prognostic value of tumor budding in muscle-invasive urothelial carcinoma of the bladder

**DOI:** 10.1186/s43046-025-00332-9

**Published:** 2025-12-01

**Authors:** Ahlem Bdioui, Asma Mahmoudi, Zaineb Lajmi, Maroua Krifa, Syrine Moussa, Oussema Belkacem, Mariem Alaya, Sarra Mestiri, Sihem Hmissa, Nabiha Missaoui

**Affiliations:** 1 Pathology Department, Sahloul University Hospital of Sousse, Sousse, Tunisia; 2 Faculty of Medicine of Sousse, Research Laboratory LR21ES03 Oncogenesis and Tumor Progression, University of Sousse, Sousse, Tunisia; 3 Biopathology Department, Institute Paoli-Calmettes, Marseille, France; 4 Pathology Department, Salah Azaiez Cancer Institute, Tunis, Tunisia; 5 Faculty of Medicine of Sousse, Research Laboratory LR21ES03 Oncogenesis and Tumor Progression, University of Sousse, Sousse, Tunisia

**Keywords:** Bladder, Muscle-invasive urothelial carcinoma, Tumor budding, Predictor, Prognosis

## Abstract

**Background:**

Bladder cancer is a common malignancy of the urogenital tract worldwide. Almost 20–30% of new cases are muscle-invasive, a complex pathology with significant clinical and biological variability. Despite treatment advances, predicting disease progression remains challenging due to the limitations of traditional prognostic factors. Identifying new markers, such as tumor budding (TB), could offer a promising breakthrough. The aim of this study was to evaluate TB in muscle-invasive urothelial carcinoma (UC) of the bladder and assess its clinicopathological and prognostic significance.

**Methods:**

We conducted a cross-sectional analytical study of 84 cases of muscle-invasive UC of the bladder. TB was assessed based on the 2016 International Tumor Budding Consensus Conference guidelines and correlated with clinicopathological features and patient survival.

**Results:**

The study included 77 men and 7 women, with a mean age of 68.13 ± 9.41 years. Histologically, ulcerative proliferative and infiltrative features were most common (58%), and high-grade tumors accounted for 92.9% of cases. Tumors were mainly classified as pT4a (40.5%), with lymph node metastasis observed in 34.3%. The mean overall survival was 23.07 ± 1.9 months. TB was present in 41.7% of cases, with Bd1 in 9.5%, Bd2 in 16.7%, and Bd3 in 15.5% of UCs. Significant correlations were found between TB and the pattern of the invasive front (*p* = 0.002), presence of carcinoma in situ component (*p* = 0.024), vascular emboli (*p* = 0.020), lymphatic emboli (*p* = 0.044), perineural invasion (*p* = 0.012), and parietal wall infiltration (*p* = 0.006).

**Conclusion:**

These findings suggest that TB serves as an independent histoprognostic predictor of the aggressiveness of muscle-invasive UC, warranting its inclusion in pathology reports and treatment protocols. However, to further validate these findings, large-scale prospective studies with standardized methods for evaluating TB are required.

## Background

Bladder cancer is the most prevalent malignant tumor affecting the urinary tract [1]. In 2022, it ranked as the 9th most prevalent cancer, with over 614,298 new cases diagnosed worldwide [2]. Among tumors of the urogenital system, bladder cancer ranks second only to prostate cancer [1, 3]. In terms of mortality, bladder cancer ranks 13th among all cancers worldwide and accounts for approximately 220,596 deaths annually, representing 2.3% of cancer-related deaths [2]. The highest incidence is observed in Europe and North America, while the lowest incidence rates are recorded in sub-Saharan Africa, Mexico, and certain Middle Eastern countries [4]. According to the Northern Tunisia National Cancer Registry, bladder cancer is the most frequent urogenital tumor in men and represents the second most commonly diagnosed cancer in men after lung cancer [5].

The epidemiology of bladder cancer varies by region, largely due to differences in exposure to risk factors such as smoking, occupational hazards, and arsenic contamination in drinking water [2]. Infection with *Schistosoma haematobium* also plays a significant role, particularly in the development of squamous cell carcinomas of the bladder [4]. The geographic variation of bladder cancer is further influenced by differences in access to diagnostic tools, such as enhanced cystoscopy [3].

At the time of diagnosis, 70–80% of bladder cancers are confined to the mucosa or submucosa, while 20–30% present as muscle-invasive urothelial carcinomas (UCs). These two categories have distinct prognoses and require different therapeutic approaches [4]. For non-muscle-invasive tumors, the standard treatment is endoscopic transurethral resection, often followed by intravesical chemotherapy to reduce the risk of recurrence, particularly in cases of primary and solitary tumors [1].

In contrast, muscle-invasive tumors are associated with poorer prognosis due to their aggressive nature and high metastatic potential. The gold standard for managing these tumors is radical cystectomy combined with pelvic lymph node dissection [1]. However, since approximately 50% of patients diagnosed with muscle-invasive tumors experience recurrence within five years following radical cystectomy, neoadjuvant cisplatin-based chemotherapy has been widely recommended, offering significant survival benefits [1, 3, 4].

To date, numerous clinicopathological factors associated with patient outcomes in muscle-invasive UC of the bladder have been reported, including lymph node involvement, distant metastases, pathological T stage (pT2–4), lymphovascular invasion, specific histological subtypes, and surgical margin status [6–8]. Among these factors, the two most significant prognostic indicators are lymph node metastases and tumor stage [6]. Nevertheless, since patients with similar clinical and pathological characteristics can exhibit diverse clinical outcomes or varied treatment responses, it is crucial to identify new biomarkers to better predict prognosis and develop novel therapeutic approaches for muscle-invasive UC of the bladder. Recently, tumor budding (TB) has been reported as a predictor of poor prognosis in various cancers, particularly colorectal cancers [9–11]. However, only a few studies have explored the prognostic insights of TB in muscle-invasive UC of the bladder [12, 13]. In this context, our study aimed to evaluate the prognostic impact of TB in muscle-invasive UC of the bladder diagnosed among Tunisian patients.

## Materials and methods

### Study design

A cross-sectional analytical study was conducted on muscle-invasive bladder UCs at the Pathology Department of Sahloul University Hospital in Sousse, Tunisia. Ethical approval was obtained from the local Human Ethics Committee at Sahloul University Hospital in Sousse (Tunisia), and the study was carried out in accordance with the principles outlined in the Declaration of Helsinki.

The study included histologically confirmed muscle-invasive UCs in patients who underwent either cystoprostatectomy or anterior pelvic exenteration between November 2019 and June 2023 at the Department of Urology, Sahloul University Hospital, Sousse (Tunisia). However, non-UC cases, carcinomas in situ (CIS) without histological evidence of invasion, tumors treated endoscopically with minimal residual tumor in the surgical specimens, extravesical (upper tract) UCs, and bladder metastases were excluded. Patients who received preoperative treatment (BCG, intravesical chemotherapy, or neoadjuvant systemic therapy) were excluded to ensure a homogeneous study population and minimize potential confounding factors.

For each patient, data were collected from clinical records, pathological reports, and the review of histological Hematoxylin and Eosin (H&E)-stained slides. The variables studied included epidemiological data (such as age, sex, and personal medical history). Pathological data included macroscopic features, such as the type of surgical specimen, tumor characteristics (size, location, appearance, consistency, unifocal or multifocal tumor, macroscopic necrosis, extension to perivesical fat) as well as microscopic features such as histological grade according to the 2022 World Health Organization (WHO) classification [14], histological subtype, stroma type, stroma abundance [15, 16], pattern of the invasive front [13], tumor-infiltrating lymphocytes (TILs) [17], microscopic necrosis, vascular emboli, perineural invasion, lymph node metastasis with or without capsular rupture, pTNM stage (according to the 8th edition of the American Joint Committee on Cancer classification [18]), and other incidental histological findings (tumors or other lesions).

### Tumor budding evaluation

For the evaluation of TB, two pathology experts reviewed all H&E slides from the relevant specimens. The assessment was performed following the recommendations of the 2016 International Tumor Budding Consensus Conference (ITBCC 2016) [19]. This consensus defined TB as the presence of isolated tumor cells or cell clusters consisting of no more than four tumor cells at the invasive front of the tumor [19]. TB was evaluated by examining all invasive fronts of the tumor and selecting the slide and area with the highest budding density. The “hotspot” method was employed to count tumor buds and was conducted independently by two blinded pathologists [19]. The assessment began with a low-magnification (*×*4 or *×*10) examination of the entire invasive tumor front to identify areas with the highest density of TB. Within these selected regions, the number of “buds” was further counted in one high-power field (×40; 22 mm lens, 0.55 mm² field area). The “hotspot” method is advantageous, as it requires evaluation of only one high power-field to assess TB, making it particularly suitable for cases with limited tissue availability or fragmented specimens. Based on the ITBCC 2016, TB was categorized as follows: Bd1 (Low TB): 0 to 4 buds; Bd2 (Intermediate TB): 5 to 9 buds; and Bd3 (High TB): >10 buds [19].

### Statistical analysis

The statistical evaluation was conducted using the Statistical Package for the Social Sciences (SPSS, version 25.0). For descriptive statistics, frequencies and percentages were calculated for qualitative variables, while means, standard deviations, and extreme ranges were determined for quantitative variables. The correlation between TB and prognostic factors was analyzed using Pearson’s Chi-square test. If the expected count was less than five, Fisher’s exact test was applied as a correction. For multivariate analysis, binary logistic regression was used for qualitative dependent variables. Odds ratios (OR) with 95% confidence intervals (CI) were calculated. Independent variables were included in the regression models if their p-value was below 0.20.

Overall survival was defined as the time (in months) from the date of diagnosis to the last follow-up or death. The date of diagnosis was determined based on transurethral bladder resection of the bladder or, if unavailable, the date of surgical intervention. Data collection was completed on December 31, 2023. Survival data were analyzed using Kaplan-Meier estimates, and comparisons were made using the log-rank test. Statistical significance was set at a p- value (p) < 0.05.

## Results

### Clinicopathological features of patients with muscle-invasive UC of the bladder

Our study cohort included 84 patients diagnosed with muscle-invasive UC of the bladder, with a mean age of 68.13 ± 9.41 years (range: 48–88 years). Patients were divided into 77 males and 7 females (male-to-female ratio: 11.0). Twenty-two patients were tobacco users. Three subjects had relevant medical histories: adenosquamous carcinoma of the colon (*n* = 1), clear cell carcinoma of the kidney (*n* = 1), and basal cell carcinoma of the nose (*n* = 1). Table 1 details the clinicopathological characteristics of patients diagnosed with muscle-invasive UC of the bladder.

**Table 1:** Clinicopathological features of patients diagnosed with muscle-invasive UC of the bladder

Characteristics	N (%)
Age (years)	
< 60	18 (21.4%)
60-70	33 (39.3%)
71-80	22 (26.2%)
>80	11 (13.1%)
Gender (n = 84)	
Male	77 (91.7%)
Female	7 (8.3%)
Antecedents (n = 84)	
Cancerous	3 (3.6%)
Non-cancerous	81 (96.4%)
Type of surgical specimen (n = 84)	
Total cystoprostatectomy	77 (91.7%)
Anterior pelvic exenteration	7 (8.3%)
Tumor size (n = 82)	
< 2 cm	9 (11%)
2 - 5 cm	39 (47.6%)
>5 cm	34 (41.5%)
Gross description (n = 69)	
Proliferative	10 (14.5%)
Polypoid	2 (2.9%)
Ulcero-proliferative and infiltrative	40 (58%)
Papillary	6 (8.7%)
Thickened wall	1 (1.4%)
Ulcerative	10 (14.5%)
Tumor focality (n = 84)	
Unifocal	37 (44%)
Multifocal	47 (56%)
Tumor consistency (n = 32)	
Friable	16 (50%)
Indurated	14 (43.8%)
Firm	2 (6.2%)
Macroscopic necrosis (n = 84)	
Present	6 (7.1%)
Absent	78 (92.9%)
Pattern of the invasive front (n = 72)	
Infiltrative	41 (56.9%)
Expansive	31 (43.1%)
Tumor grade (n = 84)	
High grade	78 (92.9%)
Low grade	6 (7.1%)
Histological subtype (n = 32)	
Squamous	23 (71.9%)
Glandular	5 (15.6%)
Plasmacytoid	2 (6.2%)
Micropapillary	2 (6.2%)
Tumor necrosis (n = 84)	
Present	53 (63.1%)
Absent	31 (36.9%)
Associated lesions (n = 73)	
Carcinoma i*n situ *component	27 (37%)
Dysplasia	11 (15.1%)
Bizarre multinucleation	3 (4.1%)
Papillary surface architecture	32 (43.8%)
Surface ulceration (n = 84)	
Present	53 (63.1%)
Absent	31 (36.9%)
Vascular emboli (n = 84)	
Present	53 (63.1%)
Absent	31 (36.9%)
Lymphatic emboli	
Present	57 (67.9%)
Absent	27 (32.1%)
Perineural invasion (n = 84)	
Present	49 (58.3%)
Absent	35 (41.7%)
Stroma abundance (n = 84)	
Moderately abundant	56 (66.7%)
Poorly abundant	19 (22.6%)
Highly abundant	9 (10.7%)
Stroma type (n = 84)	
Fibro-inflammatory	63 (75%)
Fibrous	18 (21.4%)
Inflammatory	3 (3.6%)
Surgical margin (n = 84)	
Clear	52 (61.9%)
Involved	32 (38.1%)
Pathological stage (pT) (n = 84)	
pT2	22 (26.2%)
pT3	28 (33.3%)
pT4a	34 (40.5%)
Lymph node status (pN) (n = 70)	
pN(+)	24 (34.3%)
pN0	47 (40%)
pNx	13 (18.6%)
Metastatic status (pM) (n = 84)	
pM1	2 (2.4%)
pMx	82 (97.6%)
Tumor-infiltrating lymphocytes (TILs) (n = 84)	
Low TILs	50 (59.5%)
Moderate TILs	29 (34.6%)
High TILs	5 (5.9%)
Tumor budding (n = 84)	
Absent	49 (58.3%)
Present	35 (41.7%)
Grade I	8 (9.5%)
Grade II	14 (16.7%)
Grade III	13 (15.5%)
Survival status (n = 84)	
Alive	40 (47.6%)
Dead	29 (34.5%)
Lost to follow-up	15 (17.9%)

Macroscopically, the most common tumor morphology was ulcerative-proliferative and infiltrative (58%). Tumors were unifocal in 44% and multifocal in 56% of cases. Tumor invasion was assessed in 72 cases, revealing an infiltrative pattern in 41 cases and an expansive pattern in 31 cases. Histopathologically, 92.9% of tumors were high-grade, while 7.1% were low-grade. Histological subtypes were found in 38.1% of cases, with squamous differentiation being the most common (71.9%). Tumor necrosis was present in 63.1%, associated CIS component in 37%, and dysplasia in 13.1% of UC samples. Papillary surface architecture was present in 43.8% of tumor cases. Vascular emboli were observed in 63.1%, lymphatic emboli in 67.9%, and perineural invasion in 58.3% of cases. The tumor stroma was fibro-inflammatory in 75% and moderately abundant in 66.7% of tumors. Surgical margins were involved in 38.1% of UC samples.

Tumor stage (pT) distribution was as follows: pT2 in 26.2%, pT3 in 33.3%, and pT4a in 40.5% of cases. Lymph node dissection was performed in 70 cases, revealing perivesical lymph node involvement in 33 cases. Lymph node metastases (pN+) were present in 24 tumors, including seven cases with metastatic lymph node capsule rupture. Two cases were classified as pM1 due to peritoneal carcinomatosis.

Other incidental findings included a uterine leiomyoma (*n* = 1) and a serous ovarian cystadenoma (*n* = 1) during anterior pelvic exenteration, prostatic adenocarcinoma (*n* = 12), granulomatous prostatitis (*n* = 1) during cystoprostatectomy, and renal tumor (*n* = 1), perirenal abscess (*n* = 1), and an inflammatory pseudopolyp (*n* = 1).

**Fig. 1:**
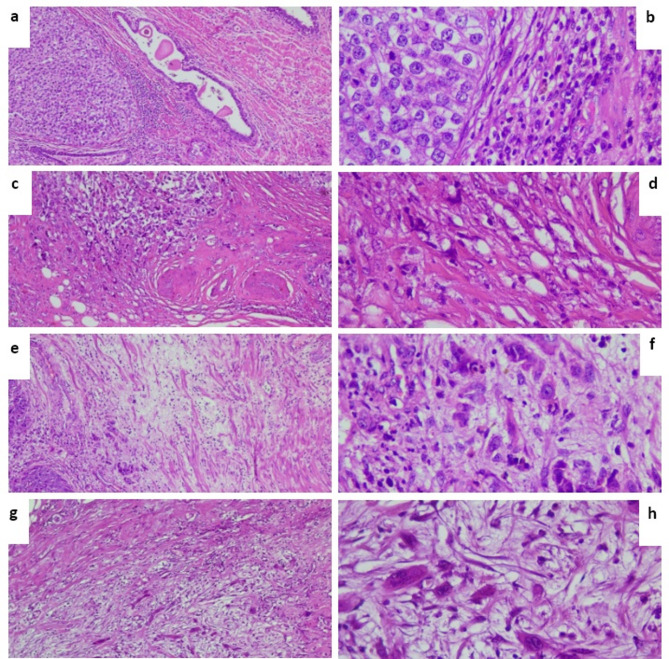
Representative histological section of different tumor budding scores observed in muscle invasive urothelial carcinoma of the bladder according to the 2016 International Tumor Budding Consensus Conference (2016 ITBCC). **a-b**: absence of tumor budding (a: x100; b: x400). **c-d**: Bd1 (Low tumor budding: 0 to 4 buds**)**: Minimal tumor budding (c: x100; d: x400). **e-f**: Bd2 (Intermediate tumor budding: 5 to 9 buds**)**: Moderate tumor budding (e: x100; f: x400). **g-h**: Bd3 (High tumor budding: >10 buds**)**: Extensive tumor budding (e: x100; h: x400)

TILs were low in 59.5%, moderate in 34.6%, and high in 5.9% of cases. Furthermore, TB was present in 41.7% of cases, with Bd1 in 9.5%, Bd2 in 16.7%, and Bd3 in 15.5% of muscle-invasive UC of the bladder (Fig. 1).

Fifteen patients were lost to follow-up. Therefore, the outcome was assessed in 69 patients. The mean follow-up duration was 14.54 ± 10.68 months (range: 1–37 months). As of December 31, 2023, the date of data collection closure, 29 patients (34.5%) had died, with a mean overall survival of 23.07 ± 1.9 months (95% CI: 19.7–27.3 months).

### Univariate analysis of TB and clinicopathological and prognostic parameters

Table 2. summarizes the univariate analysis of TB and clinicopathological parameters in patients diagnosed with muscle-invasive UC of the bladder. TB was identified in 30 of 77 male patients (39%) and 5 of 7 female patients (71.4%), with no statistically significant gender-based correlation (*p* = 0.122). The highest prevalence of TB was observed in patients aged 60–70 years (*n* = 18), followed by those aged 70–80 years (*n* = 9). However, there was no significant association between TB and age at diagnosis (*p* = 0.428).

**Table 2:** Univariate analysis: correlation of tumor budding with histoprognostic factors

Parameters		%	TB presence (%)	*P value*
Age (years)				
	<60	18 (21.4%)	8 (44.4%)	0.428
	60-70	33 (39.3%)	18 (54.5%)	
	>70	33 (39.3%)	9 (27.3%)	
Sex				
	Male	77 (91.7%)	30 (39%)	0.122
	Female	7 (8.3%	5 (71.4%)	
Tumor size				
	≤5 cm	48 (58.5%)	23 (47.9%)	0.092
	>5cm	34 (41.5%)	10 (29.4%)	
Histological grade				
	Low-grade	6 (7.1%)	2 (33.3%)	0.999
	High-grade	78 (92.9)	33 (42.3%)	
Pattern of the invasive front				
	Infiltrative	41 (56.9%)	23 (56.1%)	0.002*
	Expansive	31 (43.1%)	6 (19.4%)	
Histological subtype				
	+	32 (38.1%)	15 (46.9%)	0.448
	-	52 (61.9%)	20 (38.5%)	
Tumor necrosis				
	+	53 (63.1%)	21 (39.6%)	0.619
	-	31 (36.9%)	14 (45.2%)	
Surgical margin				
	Involved	32 (38.1%)	17 (53.1%)	0.095
	Clear	52 (61.9%)	18 (34.6%)	
TILs				
	Low TILs	50 (59.5%)	21 (42%)	0.940
	High-moderate TILs	34 (40.5%)	14 (41.2%)	
pT				
	pT2	22 (26.2%)	3 (13.6%)	0.006*
	pT3	28 (33.3%)	13 (46.4%)	
	pT4a	34 (40.5%)	19 (55.9%)	
pN				
	pN(+)	24 (28.6%)	12 (50%)	0.262
	PN(-)	47 (55.9%)	17 (36.2)	
Carcinoma* in situ* component				
	+	27 (37%)	16 (59.3%)	0.024*
	-	46 (63%)	15 (32.6%)	
Vascular emboli				
	+	53 (63.1%)	29 (54.7%)	0.020*
	-	31 (36.9%)	6 (19.4%)	
Lymphatic emboli				
	+	57 (67.9%)	28 (49.1%)	0.044*
	-	27 (32.1%)	7 (25.9%)	
Perineural invasion				
	+	49 (58.3%)	26 (53.1%)	0.012*
	-	35 (41.7%)	9 (25.7%)	

TB was present in 47.9% of tumors ≤ 5 cm and 29.4% of tumors > 5 cm (*p* = 0.092). Among high-grade tumors, 42.3% exhibited TB, compared to 33.3% of low-grade tumors (*p* = 0.999). Tumors with an infiltrative invasion front showed TB in 56.1%, significantly higher than the 19.4% observed in tumors with an expansive front (*p* = 0.002).

Histological differentiation revealed TB in 46.9% of differentiated tumors and 38.5% of pure urothelial tumors, with no significant correlation (*p* = 0.448). TB was observed in 39.62% of tumors with extensive necrosis and 45.2% of tumors without necrosis, showing no significant association (*p* = 0.619).

A significant association was found between TB and associated CIS component, with 59.3% of CIS-positive tumors exhibiting TB, compared to 33.3% of CIS-negative tumors (*p* = 0.024). TB was also significantly associated with vascular emboli (54.7% vs. 19.4%; *p* = 0.020) and lymphatic emboli (49.1% vs. 25.9%; *p* = 0.044). Additionally, TB was significantly linked to perineural invasion (53.1% vs. 25.7%; *p* = 0.012).

No significant correlation was identified between TB and surgical margin involvement (53.12% vs. 34.61%; *p* = 0.095). However, TB was significantly associated with the degree of parietal infiltration: 13.63% in pT2 tumors, 46.4% in pT3 tumors, and 55.9% in pT4a tumors (*p* = 0.006). Lymph node status showed no significant correlation with TB (*p* = 0.262). In addition, TB was observed in 42% of tumors with low TILs and 41.2% of tumors with moderate and high TILs, with no significant association (*p* = 0.940).

**Fig. 2:**
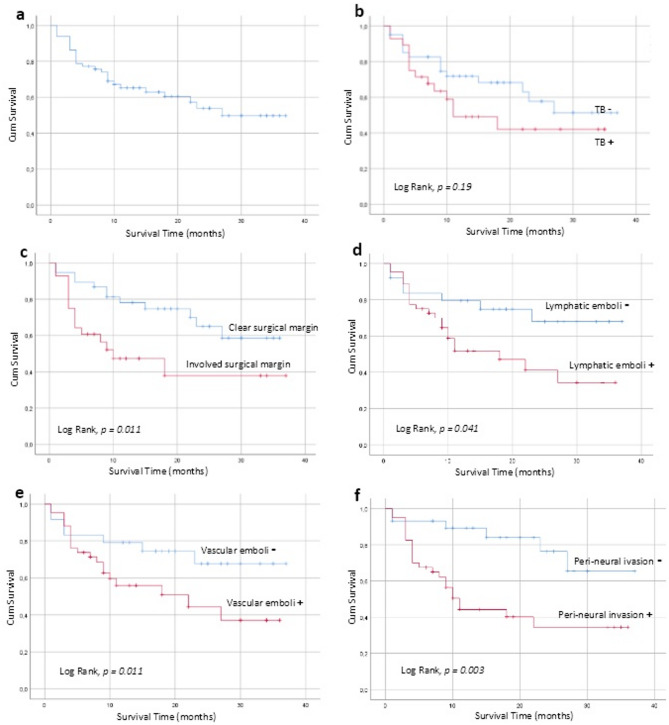
Kaplan-Meier analysis of patient survival association with tumor budding and pathological features. **a** Overall survival of patients diagnosed with muscle invasive urothelial carcinoma of the bladder. **b** No significant difference was identified, according to tumor budding (TB) status; whereas, a significant correlation was observed with surgical margin (**c**), lymphatic emboli (**d**), vascular emboli (**e**), and perineural invasion (**f**)

Overall survival was lower in patients with TB (19.05 ± 2.9 months) compared to those without (25.07 ± 2.3 months), though the difference was not statistically relevant (*p* = 0.190). However, factors significantly associated with reduced survival included surgical margin involvement (*p* = 0.011), lymphatic emboli (*p* = 0.041), vascular emboli (*p* = 0.040), and perineural invasion (*p* = 0.002) (Fig. 2). Other parameters, such as age, gender, tumor size, grade, histological subtypes, infiltration depth, lymph node status, and TB, showed a trend toward poorer survival but lacked statistical significance (*p* > 0.05).

### Multivariate analysis of TB and clinicopathological parameters

Multivariate analysis identified the pattern of the invasive front as the only independent prognostic factor significantly associated with reduced overall survival (*p* = 0.039). However, tumor size, parietal infiltration (pT), vascular and lymphatic emboli, and perineural invasion were not independent predictors of disease-free survival (Table 3).

**Table 3:** Multivariate analysis

Parameters	Odds Ratios	95% confidence intervals		*P* value
		Lower	Upper	
Perineural invasion	0.810	0.203	3.239	0.766
Pattern of the invasive front	4.290	1.078	17.073	0.039*
Tumor size	0.791	0.320	1.955	0.612
Vascular emboli	3.105	0.394	24.438	0.282
Lymphatic emboli	0.282	0.034	2.336	0.241
pT	1.493	0.613	3.635	0.378

^*^Statistically significant

## Discussion

The study evaluated the distribution of TB in muscle-invasive UC of the bladder and its correlation with clinicopathological and prognostic factors. Interestingly, TB was observed in 41.7% of tumor cases, with the following distribution: Bd1 (9.5%), Bd2 (16.7%), and Bd3 (15.5%). Furthermore, a significant correlation was found between TB and various prognostic parameters, including the degree of bladder wall infiltration, the pattern of the invasion front, the presence of vascular emboli, lymphatic emboli, perineural invasion, and the presence of an associated CIS component. To our knowledge, this study is among the few worldwide that focuses on the prognostic significance of TB in muscle-invasive UC of the bladder.

Muscle-invasive UCs of the bladder generally carry a worse prognosis compared to non-invasive forms [4]. Histopathological prognostic factors are critical in assessing the risk of progression, metastasis, and response to treatment, as well as in guiding therapeutic decisions. Epidemiological data suggest that age and sex also influence clinical outcomes. Patients over the age of 65 tend to have a poorer prognosis [8], and women experience more aggressive disease progression than men, although findings are conflicting [20]. Furthermore, the pT stage and lymph node involvement (N1-N3) are among the most significant prognostic indicators. Deeper tumor invasion correlates with a higher risk of metastasis and reduced survival rates [7, 8]. High-grade tumors and certain aggressive histological subtypes—such as squamous cell, sarcomatoid, and plasmacytoid subtypes—are linked to poorer survival outcomes and reduced responsiveness to standard therapies [6, 8]. The prognostic value of vascular and perineural invasion remains debated [8, 21]. Additionally, a positive surgical margin indicates advanced disease and is associated with compromised patient survival [22]. In our cohort, the rate of positive surgical margins was 38.1%, which is notably higher than the rates reported in the literature, typically ranging from 0% to 26% in the largest series [22, 23]. Studies focusing primarily on localized tumors (pT2) generally report low or negative margin rates. However, in cohorts that include patients with more advanced stages—such as ours—the rate of positive margins can approach 26%, as demonstrated by a large meta-analysis of 36 studies involving 10,738 patients [22]. Notably, when the study population is restricted to patients with advanced-stage disease, margin positivity can exceed 70% [24]. The high rate of positive surgical margins in our cohort can therefore be attributed to the predominance of advanced-stage tumors. This is consistent with national data from Tunisia, where studies have reported that more than 87% of patients present with pT3 or pT4 disease at diagnosis [25, 26].

Furthermore, molecular subtypes also play a role in prognosis. Luminal subtypes, characterized by epithelial biomarkers and FGFR3 mutations, are associated with better survival outcomes. In contrast, basal subtypes and p53-like tumors exhibit more aggressive behavior and are often resistant to neoadjuvant chemotherapy [27]. Additionally, immune responses within the tumor microenvironment further influence prognosis. High levels of TILs are linked to improved survival [28–31], while PD-L1 overexpression is associated with reduced survival and increased resistance to chemotherapy [31, 32].

Despite these established prognostic factors, patients with muscle-invasive UC who have similar clinicopathological characteristics can exhibit varying clinical outcomes or different responses to the same treatment [1, 4]. This discrepancy underscores the need for identifying new prognostic biomarkers that can better stratify patients into risk groups, predict their prognosis more accurately, and lead to the development of more personalized and effective adjuvant therapies and follow-up strategies. In recent years, TB has gained recognition as a valuable prognostic marker in several tumors, being associated with disease progression and worse clinical outcomes [9]. TB is a key element of the tumor microenvironment and is characterized by the presence of isolated tumor cells or small clusters of no more than four cells at the tumor invasion front. TB can also be identified within the primary tumor mass; the term “intratumoral budding” has thus been adopted to differentiate this variant from the more traditional peritumoral budding [9, 19]. Several studies have shown that intratumoral budding also has poor prognostic significance and can be associated with a more advanced pT stage, a higher risk of lymph node metastasis, and distant metastasis, as demonstrated in esophageal carcinomas and colorectal cancers, even in biopsy samples [33–37].

The TB phenomenon is closely related to the epithelial-to-mesenchymal transition (EMT), a biological mechanism in which polarized epithelial cells, typically attached to the basement membrane, undergo biochemical alterations to acquire mesenchymal cell characteristics [9, 32, 37]. This process is characterized by a reduction in intercellular contacts mediated by E-cadherin, disruption of intercellular junctions, and an increased migratory capacity, leading to enhanced invasiveness [36]. Therefore, TB is directly associated with a tumor’s ability to dissociate and migrate, reflecting its invasive potential and marking the early initiation and development of future metastases [9]. At the molecular level, Martinez-Ciarpaglini et al. [38] observed that tumor cells forming “buds” exhibit a typical EMT-related expression profile, characterized by decreased expression of miR-200a, miR-200b, and miR-200c, along with overexpression of the *ZEB1* and *ZEB2* genes.

Most studies have counted tumor buds using H&E staining in a single field (x20 magnification) that exhibits the highest density of tumor buds, a method known as the “hotspot” approach [9]. However, some studies have used multiple fields (5 or 10 at high magnification) and calculated the average [19]. Counting in multiple fields (e.g., 10 fields at x200 magnification) and then calculating the average offers the advantage of better representing the entire invasive front. Additionally, some evidence suggests improved concordance between observers with this approach. However, this method may “dilute” the average number of tumor buds in cases where they are focal but numerous [19]. The “hotspot” approach therefore better reflects the maximal TB extent at the invasive front. The ITBCC group endorses the “hotspot” method, as it is widely used in studies based on clinical outcomes and provides satisfactory concordance between observers [19]. This method also has the advantage of requiring only a single high-magnification field to evaluate the tumor buds. In other words, it is particularly useful in cases where tissue availability is limited or when samples are fragmented [9, 19].

As mentioned, TB results from the dissociation and transformation of a tumor epithelial cell into a mesenchymal cell [9, 33]. These cells have the ability to co-express both epithelial (e.g., cytokeratin) and mesenchymal (e.g., vimentin) proteins, simultaneously. It has also been revealed that the E-cadherin expression on the cell surface is reduced or even potentially absent at the tumor’s invasive front, particularly within the tumor buds of colorectal, pancreatic, endometrial, and esophageal cancers [37, 39–41]. The ITBCC group recommends counting TB on H&E-stained histological Sect [19]. However, some studies have favored counting TB using immunohistochemical staining for cytokeratin, where TB cells must exhibit cytoplasmic positivity. Accordingly, immunostaining with this antibody enhances the accuracy of TB evaluation and counting [42].

TB, extensively studied in colorectal cancer, is recognized as an independent predictor of poor prognosis, associated with high tumor grade, advanced TNM stage, lymphovascular invasion, and an elevated risk of lymph node and distant metastases [10, 12]. The prognostic significance of TB was underscored by its inclusion in the 2017 TNM classification, the 2019 WHO classification, and protocols by the American College of Pathologists and the International Collaboration on Cancer Reporting for colorectal cancer histopathology [19]. TB enhances colorectal cancer patient management in three key clinical scenarios: (1) in resected pT1 cases, it predicts lymph node metastasis risk, aiding decisions for surgical resection [43]; (2) in stage II tumors, high-grade budding is linked to shorter disease-free survival, suggesting a need for adjuvant therapy [41]; and (3) in preoperative biopsies, intratumoral budding may help identify patients for neoadjuvant therapy and predict tumor regression [44].

The prognostic insights of TB have been extended to various cancers, including those affecting the head and neck, breast, lung, esophagus, stomach, and urogenital tract [9]. Nevertheless, TB standardization as a prognostic factor in these cancers is hindered by the lack of validated staging systems and inconsistencies in methodologies across studies. In esophageal cancer, high-grade TB is linked to poor outcomes, such as lymph node metastases, advanced T-stage, and unfavorable histological features, despite variations in counting methods and thresholds [9, 41, 45]. Research, including studies by Du et al. [46], suggested that TB could guide clinical decisions, particularly in early-stage cancers, by predicting lymph node metastases and identifying candidates for radical surgeries like gastrectomy or esophagectomy with lymphadenectomy.

In pancreatic cancer, a 2019 meta-analysis of 613 patients found that high-grade TB was present in 40.9% of cases and correlated with a higher risk of recurrence and increased mortality [47]. Similarly, high-grade TB was an independent predictor of poor overall survival and a greater risk of recurrence in resected stage I-III lung adenocarcinomas [48, 49]. In breast cancer, high-grade TB is associated with lymphovascular invasion, larger tumor size, and worse survival outcomes [50, 51]. A meta-analysis of 2,341 oral squamous cell carcinomas also confirmed that high-grade TB correlates with lymph node metastases and poorer progression-free and overall survival [50]. Despite these findings, inconsistencies in defining high-grade budding and the absence of uniform evaluation criteria continue to limit its clinical application.

Only a few studies have explored the prognostic implications of TB in bladder cancer, encompassing both muscle-invasive and non-muscle-invasive UCs. In non-muscle-invasive bladder UC, Fukumoto et al. [52] reported that TB is significantly linked to T1 understaging, tumor architecture, and lymphovascular invasion. Patients with pT1 tumors that were TB-positive had a significantly lower 5-year progression-free survival rate compared to those without TB (53.8% vs. 88.4%, *p* = 0.001). TB was also independently linked to stage progression [53]. Furthermore, TB remained an independent predictor of tumor stage progression among patients who received *Bacillus Calmette-Guerin* instillation [52]. In pT1 high-grade tumors, Raventós Busquets et al. [54] found that TB correlates significantly with tumor type (papillary vs. solid) and lymphovascular invasion. The researchers proposed that TB represents a novel pathological variable that could predict tumor progression in high-grade T1 UCs and could be integrated into clinical practice. Therefore, its inclusion in the TNM classification system should be considered, as it may aid in early decision-making for radical cystectomy [54]. In muscle-invasive bladder cancer, to date, only two studies have investigated the prognostic significance of TB, both revealing significant associations with pT stage, lymph node metastases, and overall survival [54, 55].

Our study revealed no statistically significant relationship between TB and the patient’s age or sex, aligning with findings from previously published studies [54, 55]. Tumor size alone does not appear to have a prognostic impact, as the pTNM classification is primarily determined by the depth of tumor invasion (pT). However, larger tumors may indicate a longer disease progression and a potentially higher risk of complications. Nonetheless, prior studies have not identified any statistically significant correlation between tumor size and TB, which aligns with our results [54, 55]. Additionally, no statistically significant association was found between tumor location and TB, further supporting existing literature [54, 55].

No correlation was observed between TB presence and histological grade, consistent with previous findings [13, 54]. Similarly, Lorenzo et al. reported no association between TB and the presence of an associated CIS component [13]. In contrast, our study identified a statistically significant correlation between TB and the presence of a CIS component associated with the invasive tumor. This discrepancy underscores a potential area for further research and in-depth analysis to better understand its clinical and biological implications.

The extent of bladder wall infiltration by the tumor is a critical prognostic factor and plays a key role in guiding therapeutic decisions. We reported a significant correlation between TB and the depth of wall infiltration, supporting previous findings [54, 55]. The adverse impact of vascular invasion has been extensively documented in numerous studies, with their presence recognized as an independent predictor of poor prognosis, as well as an indicator of high histological grade and advanced tumor infiltration [53]. However, the correlation between TB and vascular emboli remains controversial. In our study, we identified a significant association between vascular invasion and TB, consistent with the findings of Seker et al. [55], suggesting thereby that TB may serve as a marker of aggressive tumor behavior. In contrast, lymphatic emboli have received relatively limited attention in the literature, with most studies primarily focusing on vascular emboli. Seker et al. [55] identified a link between TB and the presence of lymphatic invasion, which is consistent with our findings.

Perineural invasion is recognized as an unfavorable prognostic factor associated with an elevated risk of recurrence and distant metastasis. Unlike previous reports [54], our cohort revealed a significant association between TB and perineural invasion. Furthermore, we found a significant correlation between TB and the pattern of the invasive front, a finding not previously reported. However, no significant association was observed between TB and lymph node status, differing from the findings of earlier studies [54, 55]. While Seker et al. [55] reported a correlation between TB and both recurrence-free and overall survival, our cohort did not demonstrate a significant association between TB and overall survival.

In the multivariate analysis, the pattern of the invasive front emerged as the only independent prognostic factor significantly associated with reduced overall survival (*p* = 0.039). However, several limitations may have impacted the lack of association between tumor budding (TB) and other established prognostic indicators such as tumor grade and differentiation. These limitations include the exclusion of 15 patients due to missing follow-up data, the presence of histological subtypes in 38.1% of cases, a high proportion of pT4 tumors (40%), and a substantial rate of positive surgical margins.

Bladder UC exhibiting TB may be more resistant to conventional treatment protocols [14]. Recognizing this characteristic could influence the selection of standard neoadjuvant therapies, potentially leading to better therapeutic outcomes. Future studies should explore whether the presence of TB influences treatment response and whether specific therapeutic strategies should be considered for patients with this feature. Furthermore, if TB is confirmed as a significant prognostic factor, it could pave the way for the development of innovative therapeutic options. For example, targeted therapies or immunotherapies focused on the molecular mechanisms underlying TB could improve outcomes for high-risk patients.

Some limitations were encountered in our study. First, only muscle-invasive bladder tumors were included, as the primary objective was to assess correlations with histopathological prognostic factors, which are typically evaluable in radical cystectomy specimens—procedures generally reserved for muscle-invasive disease. However, extending this analysis to non–muscle-invasive tumors could yield valuable insights into associations with recurrence, progression, and disease-free survival. Second, suboptimal fixation of certain specimens hindered the accurate assessment of TB and tumor grade. Additionally, the lack of a standardized grading system or universally accepted threshold for bladder cancer posed challenges, as we had to rely on a grading system typically applied to colorectal cancers, given the absence of a consensus for bladder cancers. Furthermore, the unavailability of survival data affected the estimation of patient recurrence-free survival.

## Conclusion

Our findings showed a significant correlation between TB and several key histopathological parameters in muscle-invasive bladder UC. TB was associated with the degree of wall infiltration, the presence of vascular and lymphatic invasion, as well as the pattern of the invasive front—areas currently under debate in the literature. Unlike some previous studies, our results supported a significant association between TB and the presence of perineural invasion, along with an associated CIS component. No correlation was observed with patient demographics, tumor size, grade, or histological subtypes, which is consistent with prior research, except for age, which remains under discussion.

Our results suggest that TB could serve as a key predictive marker in muscle-invasive bladder UC, alongside other factors influencing patient prognosis. By integrating these findings into pathology reports, TB may provide valuable insights into tumor aggressiveness and help guide therapeutic decisions, potentially leading to more personalized clinical management. However, large-scale prospective studies employing standardized criteria for evaluating TB are necessary to validate these conclusions and explore their practical clinical application.

## Data Availability

No datasets were generated or analysed during the current study.

## Abbreviations

Bd1: Budding grade 1
CI: Confidence Intervals
H&E: Hematoxylin and Eosin
ITBCC 2016: 2016 International Tumor Budding Consensus Conference
OR: Odds Ratios
TB: Tumor Budding
TILs: Tumor-Infiltrating Lymphocytes
UC: Urothelial Carcinoma
WHO: World Health Organization
